# A high-throughput fluorescence polarization assay for discovering inhibitors targeting the DNA-binding domain of signal transducer and activator of transcription 3 (STAT3)

**DOI:** 10.18632/oncotarget.26013

**Published:** 2018-08-24

**Authors:** Po-Chang Shih, Yiwen Yang, Gary N. Parkinson, Andrew Wilderspin, Geoffrey Wells

**Affiliations:** ^1^ UCL School of Pharmacy, University College London, 29-39 Brunswick Square, Bloomsbury, London WC1N 1AX, UK

**Keywords:** STAT3, signal transducer and activator of transcription, high-throughput, DNA-binding domain, fluorescence polarization

## Abstract

Anti-cancer drug discovery efforts to directly inhibit the signal transducer and activator of transcription 3 (STAT3) have been active for over a decade following the discovery that 70% of cancers exhibit elevated STAT3 activity. The majority of research has focused on attenuating STAT3 activity through preventing homo-dimerization by targeting the SH2 or transcriptional activation domains. Such dimerization inhibitors have not yet reached the market. However, an alternative strategy focussed on preventing STAT3 DNA-binding through targeting the DNA-binding domain (DBD) offers new drug design opportunities. Currently, only EMSA and ELISA-based methods have been implemented with suitable reliability to characterize STAT3 DBD inhibitors. Herein, we present a new orthogonal, fluorescence polarization (FP) assay suitable for high-throughput screening of molecules. This assay, using a STAT3^127-688^ construct, was developed and optimized to screen molecules that attenuate the STAT3:DNA association with good reliability (Z’ value > 0.6) and a significant contrast (signal-to-noise ratio > 15.0) at equilibrium. The assay system was stable over a 48 hour period. Significantly, the assay is homogeneous and simple to implement for high-throughput screening compared to EMSA and ELISA. Overall, this FP assay offers a new way to identify and characterize novel molecules that inhibit STAT3:DNA association.

## INTRODUCTION

**Figure 1:**
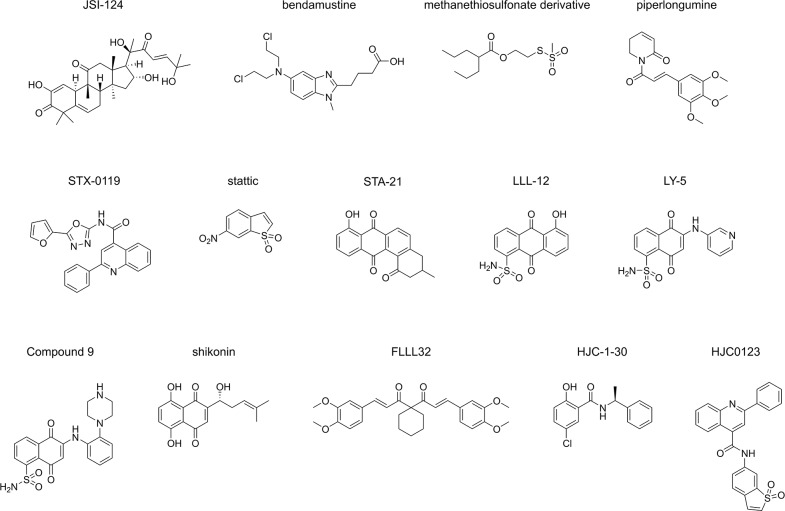
STAT3 dimerization inhibitors Published STAT3 dimerization inhibitors validated by at least one of the following assays: FP assay, AlphaScreen™ assay, cytoblot assay, FRET assay, SPR assay and ELISA.

STAT3, signal transducer and activator of transcription 3, is a key component in several signalling pathways [1, 2], is over-activated in approximately 70% of cancers [3] and plays critical roles in cell proliferation, cell survival, angiogenesis, immune invasion and metastasis [4]. Intensive efforts have been made over more than a decade to discover and develop small-molecule inhibitors to abate STAT3 activity through targeting its SH2 or transcriptional activation domains for the lowering of dimerization and activation of the protein [5–17]. During the discovery of STAT3 dimerization inhibitors, numerous cell-free and cell-based assays were developed to validate the inhibitory effect of selective inhibitors on dimerization. These include assays based on high-throughput fluorescence polarization (FP) [10, 13, 15, 18], AlphaScreen™ [6, 7, 19], fluorescence resonance energy transfer (FRET) [6, 9, 20], enzyme-linked immunosorbent assays (ELISA) [21], cytoblot [5, 22, 23], and surface plasmon resonance (SPR) methodologies [8]. The FP, AlphaScreen™, ELISA and SPR assays are all applicable to high-throughput screening and are cell-free, while the remaining techniques are also applicable to high-throughput approaches, and are cell-based. The application of these assays as screening platforms resulted in the discovery and validation of STAT3 dimerization inhibitors JSI-124 [5], bendamustine [6], piperlongumine [8] and static [10]. Moreover, the assays were also utilized as tools to support the discovery of other STAT3 dimerization inhibitors such as STX-0119 [9], STA-21 [11] and LLL-12 [12] which were all initially identified by *in-silico* high-throughput screening, and additionally applied to LY5 [13], shikonin derivatives [14], “Compound 9” [15], HJC-1-30 [24] and HJC0123 [16] and FLLL32 [17] (Figure 1) which were designed based on previously published chemical structures.

An understanding of the pathway for STAT3 activation and the individual roles and functions of each STAT3 domain allows the targeting and subsequent attenuation of STAT3 activity in a specific and selective manner. STAT3 consists of six domains with different functions in the signal transduction pathway. The domain organization of the protein from the N- to C-terminus is as follows: the N-terminal domain (ND) which mediates the tetramerization of two STAT3 dimers when binding to the promoters of target genes [25, 26]; the coiled-coil domain responsible for interacting with other cytoplasmic proteins [27]; the DNA-binding domain (DBD) through which STAT3 binds to the promoter sequences of genes [28]; the linker domain which lies between the DNA-binding and Src homology 2 (SH2) domains; the SH2 domain which plays a role in dimer formation with another phosphorylated STAT3 monomer (via phosphotyrosine residue(s), (pY) in the transcriptional activation domain) for initial binding of STAT3 to DNA [29, 30]; and the transcriptional activation domain (TAD) at the C-terminus which includes the pY site(s) for facilitating STAT3 dimerization and also is involved in the interactions with other nucleoplasmic proteins for the activation of transcription [31].

**Figure 2:**
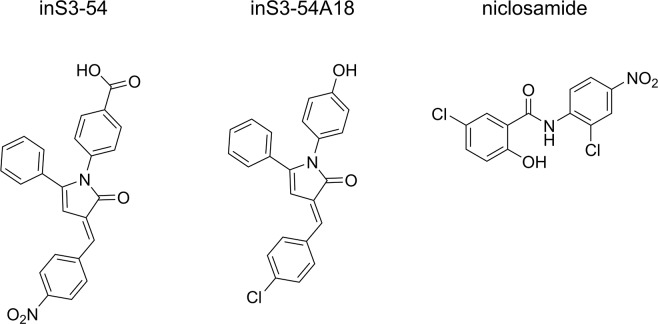
STAT3 DBD inhibitors Published STAT3 DBD inhibitors validated by at least one of the following assays: EMSA and ELISA.

Although one STAT3 dimerization inhibitor (C188-9) has advanced to early-phase clinical studies, it did not progress beyond this point [32], suggesting that preventing STAT3 dimerization through targeting the SH2 domain or TAD might be an intractable approach. Therefore, we and others have focused on inhibiting STAT3 DNA-binding through targeting the DBD. The small-molecule STAT3 DBD inhibitor (inS3-54) was reported in the literature in 2014, using an EMSA-based assay to determine inhibition of DNA-binding [33]. Other small-molecule STAT3 DBD inhibitors reported subsequently include additional inS3-54 analogues [34], and niclosamide which was validated using ELISA [35] (Figure 2). Of the two approaches used in these studies, only ELISA is applicable to high-throughput screening of compounds. Therefore, the development of a new orthogonal assay for discovering STAT3 DBD inhibitors would be desirable. Herein, we present an optimized high-throughput applicable FP assay for monitoring the STAT3:DNA association, referred to as the STAT3^127-688^:DNA FP assay. In brief, this assay uses a soluble STAT3^127-688^ protein and a Bodipy-DNA conjugate as the fluorescent probe: the latter can be displaced by competitor ligands introduced during the experiment. The protocol is simple to implement compared to EMSA and ELISA, and there are no immobilised assay components, no addition of antibodies is required, and no washing procedures are involved, all of which impact on the time, cost and reliability of the assay.

## RESULTS

### Optimized preparations: STAT3^127-688^ target protein, and the Bodipy-DNA conjugate

**Figure 3:**
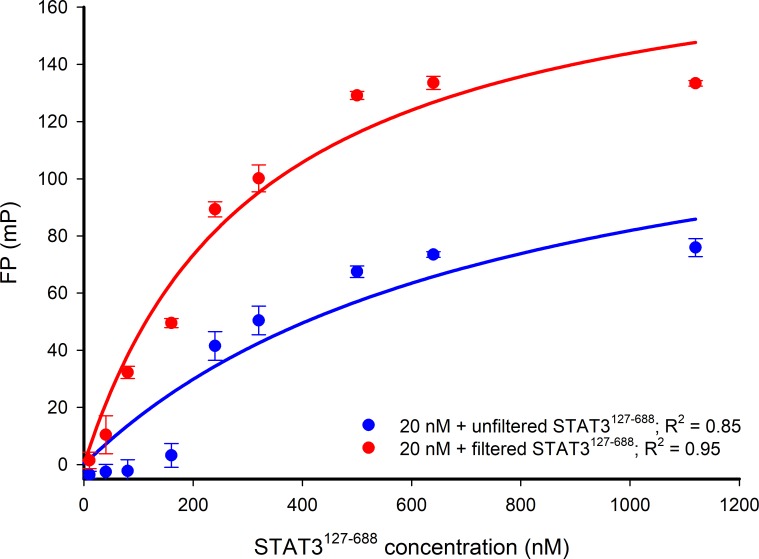
FP versus STAT3^127-688^ concentration-response curves for 20 nM Bodipy-DNA conjugate applied with either filtered or unfiltered STAT3^127-688^ The response curve obtained by using unfiltered STAT3^127-688^ shows that low FP signals are detected when applying STAT3^127-688^ concentrations lower than 200 nM. The filtration of STAT3^127-688^ (300 kDa molecular weight cut-off filter) gave more consistent FP signal responses below 200 nM STAT3^127-688^ and an improved maximum FP signal. The NaCl concentration in each experiment was < 200 μM. R^2^ represents the coefficient of determination.

To prepare the STAT3^127-688^ protein, an *E. coli* Rosetta strain was transformed with a recombinant pET-32a(+) plasmid containing the required STAT3 sequence (encoding residues 127 to 688) lacking the ND and TAD. The expressed crude protein was isolated and stored at -20°C as pellets from ammonium sulphate precipitation. The crude protein was purified using ion-exchange chromatography and the purified STAT3^127-688^ was stored in the elution buffer (~200 mM NaCl, 1 mM dithiothreitol (DTT), 25 mM Tris pH 8.5). The conditions utilized in the subsequent STAT3^127-688^:DNA FP assays require the lowering of the salt and DTT concentrations by diafiltration using a 50 kDa concentrator to a final NaCl concentration < 200 μM. The purified protein was examined by SDS-PAGE and found to be composed of a single component with a molecular weight consistent with that expected for the construct (Supplementary Figure 1A). An additional centrifugation step using a 300 kDa centrifugal filter removed misfolded, unfolded or aggregated STAT3^127-688^ that may impact on DNA binding [36], and provided a protein that gave more consistent FP responses (Figure 3).

The Bodipy-DNA conjugate was purchased in the form of two complementary single-stranded DNA (ssDNA) sequences that were annealed in a salt- and DTT-free buffer (25 mM Tris pH 8.5). Bodipy 650/665 was selected as the fluorophore due to its relative insensitivity to pH changes and long absorption and emission wavelengths, which reduces the potential for fluorescence interference derived from aromatic small-molecule inhibitors of the STAT3:DNA association.

### Optimization assays: 20 nM Bodipy-DNA conjugate and 480 nM STAT3^127-688^

**Figure 4:**
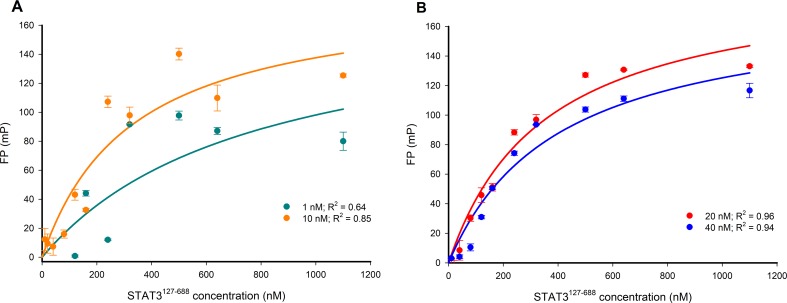
FP versus STAT3^127-688^ concentration-response curves for varying concentrations of the Bodipy-DNA conjugate **(A)** 1 and 10 nM Bodipy-DNA conjugate; **(B)** 20 and 40 nM Bodipy-DNA conjugate. 20 nM was selected as the optimal Bodipy-DNA concentration.

The optimal working concentration of the Bodipy-DNA conjugate was determined in four separate FP experiments using either 1 nM, 10 nM, 20 nM, or 40 nM of the probe for titration against increasing STAT3^127-688^ concentrations. When the data were fitted to a one site saturation binding model, a good fit was observed for the 20 nM and 40 nM Bodipy-DNA concentrations, but not for 1 nM and 10 nM concentrations (Figure 4). Accordingly, a 20 nM Bodipy-DNA concentration and a STAT3^127-688^ concentration of 480 nM (which gave 80% of the maximum FP response [37]) were selected for use in subsequent competition assays.

### Competition experiments with unlabelled DNA sequences

**Figure 5:**
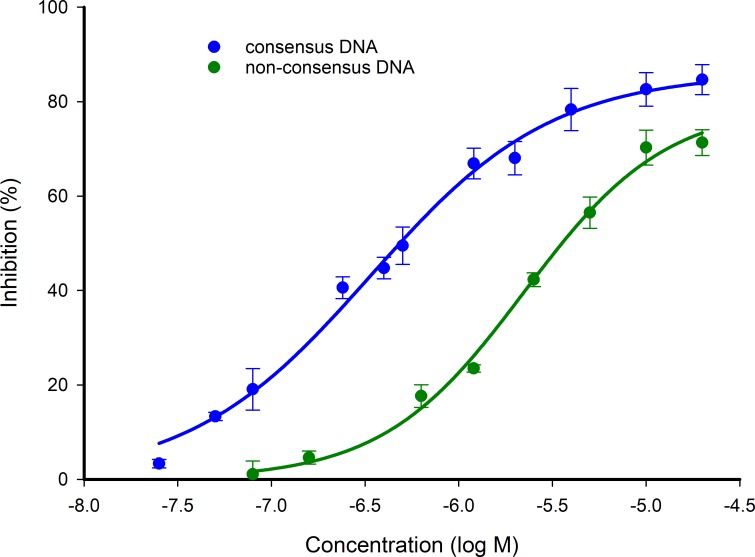
Competitive inhibition of Bodipy-DNA-STAT3^127-688^ binding by consensus and non-consensus DNA sequences The non-consensus DNA (self-complementary 5’-GTACCATGGTAC-3’) bound to the protein with a lower affinity than the consensus DNA (5’-ATTTCCCGTAAA-3’ and 5’-TTTACGGGAAAT-3’). Measurements were made after a 24 hr incubation at 4°C.

To assess the displacement of the Bodipy-DNA conjugate, a 12-mer (12 base pair) unlabelled non-consensus DNA sequence or a 12-mer unlabelled consensus DNA sequence (identical to that of the Bodipy-DNA conjugate) were applied as competitive inhibitors to displace the 12-mer Bodipy-DNA conjugate from STAT3^127-688^. The half-maximal inhibitory concentration (IC_50_) of the consensus DNA was determined as 0.30 ± 0.20 μM, while the non-consensus DNA gave an IC_50_ of 2.3 ± 0.66 μM after 24 hr (Figure 5). Although the non-consensus DNA was not expected to bind to STAT3^127-688^, the result shows that there was an association but with a weaker binding affinity. This could be explained by non-specific binding due to electrostatic interactions between the DNA backbone and STAT3. This is consistent with previous observations that transcription factors can interact non-specifically with non-consensus sequences with lower binding affinities [38, 39].

### The STAT3:DNA association is time-dependent

**Figure 6:**
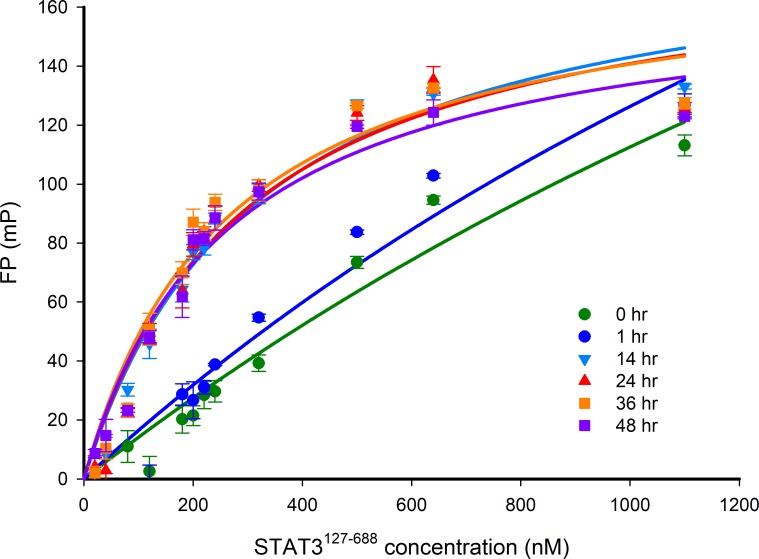
FP versus STAT3^127-688^ concentration-response curves after different incubation times The FP signal was measured after incubation of 20 nM Bodipy-DNA conjugate and varying concentrations of STAT3^127-688^ for between 0 and 48 hr. The response curves show that the STAT3:DNA association reached equilibrium after 14 hr. A STAT3^127-688^ concentration of 480 nM gave 80% of the maximum FP response, which was selected for use in competition experiments.

Before introducing small-molecule inhibitors into the assay, it is essential to understand the kinetics of the interaction between STAT3^127-688^ and the Bodipy-DNA conjugate. Therefore, the equilibrium association of STAT3^127-688^ (480 nM) and Bodipy-DNA (20 nM) was monitored after various incubation times. Our results show that after 14 hours, an equilibrium was reached that was stable for at least 48 hr (Figure 6). The length of time to reach equilibrium indicates slow binding kinetics between the two binding partners.

### Reliability and signal-to-noise: Z’ values and S:N ratios

The suitability of the STAT3^127-688^:DNA FP assay for high-throughput screening was assessed by measuring the Z’ value (values 1 > Z’ ≥ 0.5 indicate a reliable assay [40]) and the signal-to-noise (S:N) ratio were determined at various incubation times (Table 1). Caution is required with interpreting the values of Z’ and S:N ratios after short incubation times (0 and 1 hr) as equilibrium is achieved after ≥ 14 hr. However, values determined after 14 hr are considered to be sufficiently robust to monitor competition between the Bodipy-DNA conjugate and inhibitors for the association with STAT3^127-688^.

**Table 1:** Calculated Z’ values and S:N ratios at various incubation times

Time course	Z’ values	S:N ratios
0 hr	0.53	9.16
1 hr	0.71	27.39
14 hr	0.63	20.06
24 hr	0.71	19.65
36 hr	0.72	19.16
48 hr	0.74	15.78

### Validation of the assay: inS3-54, inS3-54A18 and niclosamide are inhibitors of the STAT3:DNA association

**Figure 7:**
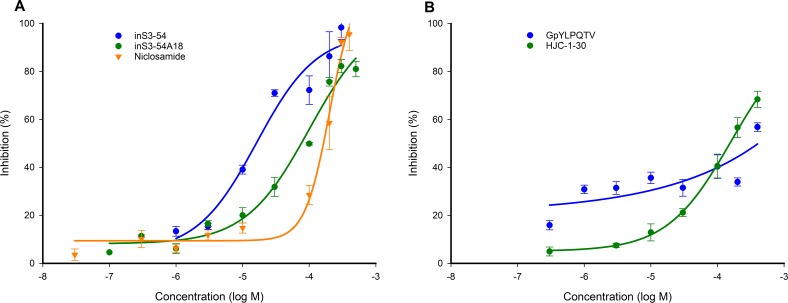
Validation of the STAT3^127-688^:DNA FP competition assay using published STAT3 inhibitors Percentage inhibition of Bodipy-DNA binding versus inhibitor concentration response curves for **(A)** STAT3 DBD inhibitors inS3-54, inS3-54A18 and niclosamide and **(B)** STAT3 dimerization inhibitors GpYLPQTV and HJC-1-30. Measurements were made after a 24 hr incubation at 4°C.

To understand the applicability of the STAT3^127-688^:DNA FP assay in compound screening campaigns, a series of pilot competition studies were conducted with known STAT3 inhibitors. The STAT3 DBD inhibitors, inS3-54, inS3-54A18 and niclosamide were tested along with STAT3 dimerization inhibitors, GpYLPQTV and HJC-1-30. GpYLPQTV is a peptide sequence derived from the gp130 subunit reported to bind to the STAT3 SH2 domain [18]. The activities of inS3-54, inS3-54A18 and niclosamide were found to be dose- and time-dependent (time-dependent data not shown) as previously described [33–35]. Estimated IC_50_ values determined for inS3-54, inS3-54A18 and niclosamide were 21.3 ± 6.9 μM, 126 ± 39.7 μM and 219 ± 43.4 μM after 24 hr respectively (Figure 7). On the other hand, GpYLPQTV and HJC-1-30 were poor inhibitors of the STAT3:DNA association achieving only 50% and 70% inhibition respectively at 400 μM after incubation for 24 hr (Figure 7).

### The IC_50_ determination of the STAT3^127-688^:DNA FP assay is comparable to the protein electrophoretic mobility shift assay (PEMSA)

In order to confirm the binding behavior of selected ligands to the STAT3^127-688^ construct, we expressed and purified a recombinant yellow fluorescent protein YFP-STAT3^127-688^ fusion protein in the same manner as that for STAT3^127-688^ which were adapted from the method used for a STAT3^127-722^ construct as previously described [41] (see Supplementary Materials and Methods). The purity of YFP-STAT3^127-688^ is shown in Supplementary Figure 1B. A protein electrophoretic mobility shift assay (PEMSA) [41], that is under further development in our labs using YFP-STAT3^127-688^ and the 12-mer unlabelled consensus DNA for STAT3, was carried out to give estimated IC_50_ values for the inhibitors. The values for inS3-54 and inS3-54A18 were ~26 μM and ~165 μM, respectively, after 24 hr incubation at 4°C (Supplementary Figures 2 and 3), which are comparable to those determined by the FP assay (21.3 ± 6.9 μM, 126 ± 39.7 μM respectively).

## DISCUSSION

Since STAT3 is highly involved in oncogenic pathways, there has been significant research directed towards finding inhibitors that interact with STAT3 or its upstream effectors. Identification of compounds that inhibit STAT3 dimerization has been a major focus. C188-9 has progressed to early-stage clinical trials [32], although it did not advance beyond this point. Recently, there has been an increased focus on blocking the interaction between STAT3 and DNA; however, the lack of direct binding assays has hindered structure-based drug design efforts in this area.

Consequently, we have developed a fluorescence polarization method to quantify STAT3 DNA-binding using a double-stranded DNA-fluorophore conjugate that has the consensus sequence 5’-TTNCNNNAA-3’[42] tethered to Bodipy at the 5’ end. The assay utilizes a STAT3^127-688^ construct, which lacks the Tyr705-containing transactivation domain (TAD) that is involved in phosphorylation-mediated dimerization of STAT3 and omits the N-terminal domain (ND) that is involved in tetramerization. Thus, the assay should be useful for identifying compounds that inhibit the STAT3:DNA association via binding to non-TAD/ND sites. The assay is a cell-free alternative to the EMSA and ELISA that have been applied previously in STAT3 DNA-binding inhibitor screening studies [33, 35]. This means that effects on upstream proteins or off-target effects on STAT3 cellular concentrations can be ruled out. The use of [^32^P] end-labelled DNA, as is the case with EMSA, is avoided, and the use of recombinant STAT3 protein removes the need for cell nuclear extracts as a source of STAT3 protein which was utilized in the cell-based ELISA protocol as described by Furtek et al. [35].

The FP assay has some advantages in terms of throughput compared to ELISA and EMSA-based methods. Unlike EMSA, the FP-based experiments can be carried out in microtiter plates and are amenable to parallelization and automation. The assay is homogeneous unlike the ELISA method that requires washing steps and the measurement is direct, in contrast to ELISA that requires antibody-mediated amplification of the readout. Additionally, the STAT3^127-688^:DNA FP assay described here, and the related STAT3-phosphopeptide FP technique utilizing FITC-GpYLPQTV as an SH2 domain-interactive probe (adapted from the method of Schust & Berg) [18] can be performed concurrently in two halves of the same 96-well black microtiter plate using suitable optical modules for each labelled probe (data not shown). Such an approach allows comparisons of the displacement of DNA and phosphopeptide fluorescent probes to be determined using the same protein construct, potentially allowing DBD and STAT3 dimerization inhibitors to be distinguished.

The applicability of the STAT3^127-688^:DNA FP assay for high-throughput screening was validated using previously described STAT3 DBD and dimerization inhibitors (Figures 5 and 7). IC_50_ values determined after 24 hr were: inS3-54, 21.3 ± 6.9 μM; inS3-54A18, 126 ± 39.7 μM; and niclosamide, 219 ± 43.4 μM (Figure 5). The binding affinities of inS3-54 and S3-54A18 were further validated using the YFP-STAT3^127-688^:DNA PEMSA that is under further development in our laboratories (Supplementary Figures 2 and 3). These values are not entirely consistent with those reported using cell-based assays using either the STAT3-dependent luciferase reporter assay (IC_50_ ~14 μM for inS3-54 and ~11 μM for inS3-54A18) or the ELISA-based method (IC_50_ ~0.2 μM for niclosamide) [32, 33, 35]. This raises the possibility that inS3-54 and inS3-54A18 may have additional activities in cells that contribute to the inhibition of STAT3-dependent luciferase reporter activity that warrant further investigation. A similar rationale may apply to niclosamide-treated HeLa cells from which nuclear extracts are taken for use in the ELISA-based detection of STAT3 activity [35].

The data support the use of the assay for characterizing both small molecule- and DNA-based competitors; the latter may be useful for quantifying the binding affinity of various consensus STAT3 binding sequences (Figures 5 and 7). The reliability of this FP assay was verified by the calculation of Z’ values and S:N ratios at time points up to 48 hr (Table 1). Refinement of the time-dependent activities of STAT3 inhibitors is possible; however, the slow association kinetics of STAT3^127-688^ and the Bodipy-DNA probe would need to be considered when carrying out such experiments (Figure 6).

Taken together, our STAT3^127-688^:DNA FP assay is a useful addition to the available assays for discovering STAT3 DBD inhibitors, because this assay is: 1) applicable to high-throughput screenings using both small molecule-based and nucleic acid-based libraries in a multi-well plate format, 2) suitable for the validation of the inhibitory effect on the STAT3:DNA binding for inhibitors, 3) able to determine dose- and time-dependent activities of STAT3 DBD inhibitors, 4) simple to conduct in comparison with the ELISA and EMSA methods, and 5) can be performed in parallel with the STAT3-phosphopeptide FP assay for discovering dimerization inhibitors on the same plate. In principle the assay methodology could be applied to other members of the STAT family of transcription factors including STAT1 and STAT5; both proteins have been cloned and expressed, and STAT1 has been co-crystallized with DNA facilitating the design of equivalent protein constructs for FP assays [43–45].

## MATERIALS AND METHODS

### Preparation of inS3-54, inS3-54A18, niclosamide, GpYLPQTV and HJC-1-30

Both inS3-54 and inS3-54A18 were synthesized using methods adapted from those described by Huang et al. [34]. The chemical structures of inS3-54 and inS3-54A18 were confirmed by ^1^H NMR, ^13^C NMR and LC/MS and analytical purities of >95% were recorded (^1^H NMR and LC/MS). Niclosamide and GpYLPQTV were purchased in a powder form from Sigma-Aldrich and Generon, respectively. HJC-1-30 was provided by Dr Charlie Nichols. The inhibitors were dissolved in 100% DMSO to form stock solutions for the STAT3^127-688^:DNA FP assay.

### Oligonucleotides

All oligonucleotide sequences were purchased from Eurofins as freeze-dried powders. The sequence of the unlabelled non-consensus DNA was self-complementary 5’-GTACCATGGTAC-3’. The sequences of the Bodipy-DNA conjugate 5’-ATTTCCCGTAAA-3’ and 5’-TTTACGGGAAAT-3’ and the unlabelled consensus DNA are identical. The fluorophore, Bodipy 650/665, was chemically linked to the 5’ end of 5’-ATTTCCCGTAAA-3’. The Bodipy-DNA conjugate sequence was designed based upon M67 core sequence 5’-TTCCCGTAA-3’[46], while the unlabelled non-consensus DNA was designed to minimise similarity with the STAT3 consensus sequence 5’-TTNCNNNAA-3’ [42].

### DNA annealing

The oligonucleotides were dissolved in the annealing buffer (25 mM Tris pH 8.5). An equal volume and concentration of each ssDNA was mixed in an Eppendorf tube that was then incubated at 95°C for 3 min, followed by cooling to room temperature overnight (in a 1 kg heatblock). This double-stranded DNA was then stored at -20°C. The Bodipy-DNA conjugate was prepared in the dark to prevent photoquenching of the fluorophore.

### STAT3^127-688^ expression and purification

STAT3^127-688^ expression was conducted as previously described [41]. One day before the STAT3^127-688^:DNA FP assay was performed, the ammonium sulphate-precipitated crude protein pellet was re-suspended using a solution of 1 mM DTT 25 mM Tris pH 8.5 and purified using HiTrap QFF columns (GE Healthcare) coupled to a fast protein column chromatography (FPLC) instrument (NGC™ Chromatography Systems, Bio-Rad). The elution of the STAT3^127-688^protein was conducted using a solution of 1 mM DTT and 25 mM Tris pH 8.5, and a gradient of increasing NaCl concentration. The purified STAT3^127-688^ usually eluted at 200 mM NaCl and was collected in a 96-well block. The purified STAT3^127-688^ was stored in the eluent at 4°C. The purity of STAT3^127-688^ was determined using SDS-PAGE.

### Preparation of the STAT3^127-688^ sample for the STAT3^127-688^:DNA FP assay

On the day the STAT3^127-688^:DNA FP assay was performed, the STAT3^127-688^ protein sample was prepared immediately for use, usually at 0.4 mg/ml. Buffer exchange was performed at least three times by diafiltration conducted using a 50 kDa centrifugal concentrator (Sartorius). The residual STAT3^127-688^ protein solution was diluted 10-fold with diafiltration buffer, 25 mM Tris pH 8.5, upon completion of each diafiltration step. Aggregated STAT3^127-688^ was removed using a benchtop centrifuge if aggregation was observed on visual inspection of the samples. Finally, invisible STAT3^127-688^ aggregate was removed from the sample by centrifugation through a 300 kDa centrifugal filter (Sartorius) at ~800 g.

### Conduct of the STAT3^127-688^:DNA FP assay

The FP assay was conducted in 96-well Chimney Well black microtiter plates (Greiner Bio-One). The final concentration of FP buffer was composed of 5% glycerol, 20 mM Tris pH 8.5, 1 mM EDTA, 0.01 mg/ml bovine serum albumin, 4% DMSO, with or without 480 nM purified STAT3^127-688^, 20 nM Bodipy-DNA conjugate, with or without a variable concentration of inhibitor, to make a total volume of 100 μl per well. After the addition of all assay components, the plates were incubated and gently agitated for the first hour at room temperature, followed by further incubation at 4°C. During incubation, the plates were covered with black lids to protect the samples from light and to reduce evaporation of the FP buffer solution. The plates were read at various incubation times using an FP plate reader (PHERAstar, BMG Labtech) with an FP 590-675-675 optical module (BMG Labtech).

### Calculations of Z’ value and S:N ratio

The Z’ values were calculated using the equation Z’ = 1 - (3SD_bound_ + 3SD_free_)/(mean of mP_bound_ – mean of mP_free_), where the SD is the standard deviation of the measurements and the mP is the measured fluorescence polarization [40]. The mP was automatically calculated by the FP plate reader using the equation mP = (I_║_−I_┴_)/(I_║_+I_┴_) where I_║_ and I_┴_ are the fluorescence polarization intensities of the Bodipy-DNA conjugate with polarizations parallel and perpendicular to the incident light, respectively. The bound state was determined by incubating 20 nM Bodipy-DNA conjugate with 480 nM STAT3^127-688^ protein, whereas for the free state, the same mixture was incubated with an additional 10 μM of unlabelled consensus DNA as a competitor. The equation S:N = (mean of mP_bound_ – mean of mP_free_)/(SD_bound_^2^ + SD_free_^2^)^0.5^ was utilized to determine the signal-to-noise ratios [47].

### Analytical methodology

Each assay condition was evaluated with at least three repeats. Binding curves were fitted using SigmaPlot 13 using the ‘one site saturation’ and ‘sigmoidal dose-response (variable slope)’ non-linear regression curve-fitting functions. The ‘one site saturation’ model uses the equation y = (B_max_x)/(K_D_+ x), where B_max_ is the maximal FP response and K_D_ is the dissociation constant. The ‘sigmoidal dose-response (variable slope)’ utilizes the equation y = min + (max – min)/(1 + 10^b(logIC50 - x)^), where max and min represents the maximum and minimum response plateaus respectively, and b is the Hill slope for the binding event. The error bars shown in the graphs are SDs from the mean values of the replicates.

## SUPPLEMENTARY MATERIALS FIGURES AND TABLES



## Abbreviations

12-mer: 12 base pair
DBD: DNA-binding domain
DTT: dithiothreitol
ELISA: enzyme-linked immunosorbent assay
EMSA: electrophoretic mobility shift assay
FP: fluorescence polarization
FRET: fluorescence resonance energy transfer
ND: N-terminal domain
PEMSA: protein electrophoretic mobility shift assay
pY: phosphorylated tyrosine
SD: standard deviation
SH2: SRC homology 2
S:N: signal-to-noise ratio
SPR: surface plasmon resonance
STAT3: signal transducer and activator of transcription 3
STAT3^127-688^: a truncated STAT3 with residues 127-688
TAD: transcriptional activation domain.
