# Supplementary material for: A high-throughput fluorescence polarization assay for discovering inhibitors targeting the DNA-binding domain of signal transducer and activator of transcription 3 (STAT3)
Source: Oncotarget. 2018 Aug 24;9(66):32690–701. doi: 10.18632/oncotarget.26013 (PMC6135694; doi:10.18632/oncotarget.26013)
Supplement: Supplementary file 1 [file oncotarget-09-32690-s001.pdf]

A high-throughput fluorescence polarization assay for discovering inhibitors targeting the DNA-binding domain of signal transducer and activator of transcription 3 (STAT3)

SUPPLEMENTARY MATERIALS

The recombinant pET-32a(+) plasmid containing the STAT3$^{127-688}$ gene has the same STAT3 sequence as the plasmid including a yellow fluorescent protein YFP-STAT3$^{127-688}$. The recombinant fusion protein YFP-STAT3$^{127-688}$ was expressed and purified using the methods adapted from those for STAT3$^{127-688}$. The purity of YFP-STAT3$^{127-688}$ is shown in Supplementary Figure 1B.

SUPPLEMENTARY METHODS

PEMSA method

The ion-exchange purification of YFP-STAT3$^{127-688}$ protein required elution into a buffer containing ~400 mM NaCl, 25 mM Tris, 1 mM DTT, pH 8.5. Purity was shown by SDS-PAGE (Supplementary Figure 1). Incubation of the protein with inhibitors and the 12-mer unlabelled DNA was carried out using procedures identical to those for STAT3$^{127-688}$. The PEMSA was carried out using a method adapted from Nkansah et al. [41]. Briefly, samples were prepared with a mixture of a binding buffer (20 mM HEPES, 0.03 mM EDTA, 1 mM $MgCl_2$, 40 mM KCl, 0.2 mg/ml BSA and 8% glycerol), the 12-mer unlabelled consensus DNA (6 µM) and the purified YFP-STAT3$^{127-688}$ (0.8 µM), and incubated for 24 hr at 4°C. 5% native polyacrylamide (37.5:1 acrylamide:bis ratio) gels (20 mM Tris, pH 8.3, 18 mM boric acid, 2 mM EDTA and 1% glycerol) were pre-run with running buffer (25 mM Tris, pH 8.3, 22.5 mM boric acid and 2.5 mM EDTA) at 150 volts at 4°C for 2 hr. After that, the incubated samples were loaded onto the gels and run at 150 volts at 4 °C for 70 min. The results were visualized using a Safe Imager Transilluminator, and the analyses were carried out using ImageJ and SigmaPlot 13.

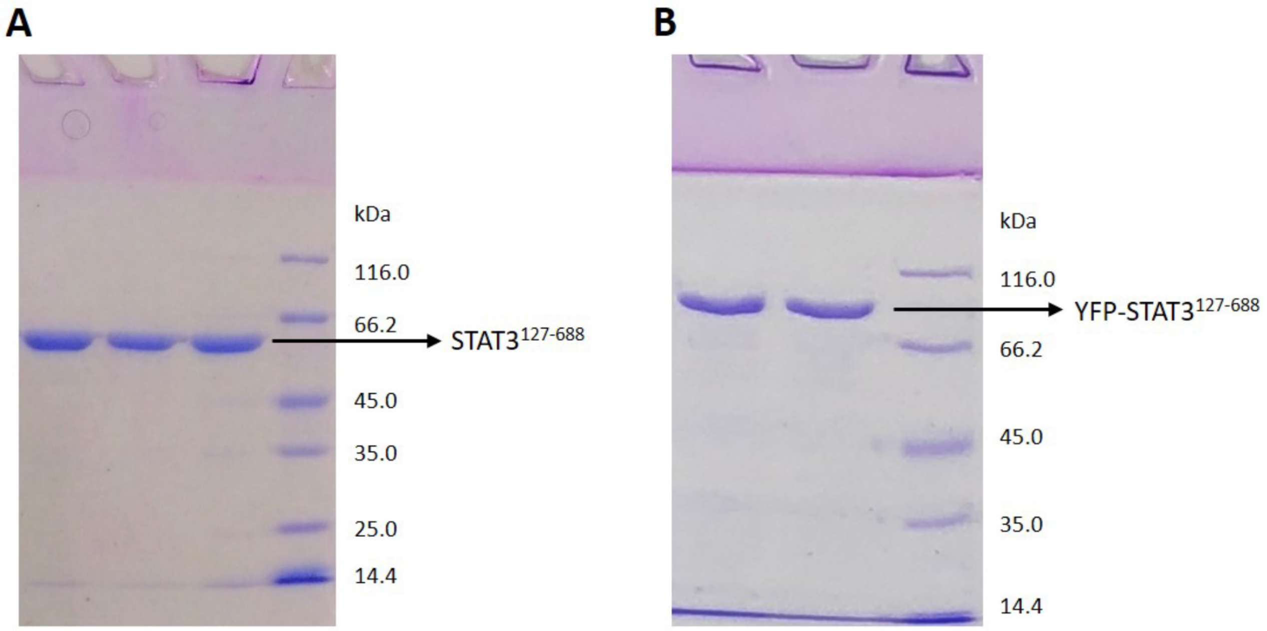

Supplementary Figure 1: Purity of STAT3$^{127-688}$ and YFP-STAT3$^{127-688}$. (A) The purity of the STAT3$^{127-688}$ (lanes 1-3) was verified using SDS-PAGE. **(B)** The purity of the YFP-STAT3$^{127-688}$ (lanes 1-2) was verified using SDS-PAGE.

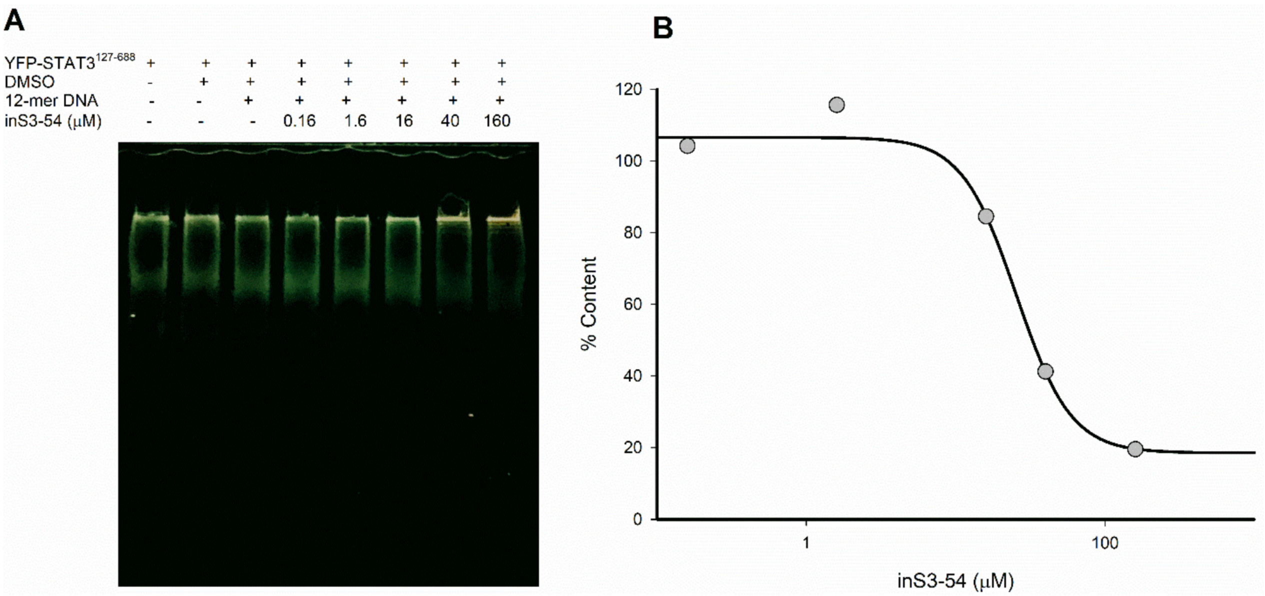

Supplementary Figure 2: Validation of the inhibitory effect of inS3-54 on YFP-STAT3$^{127\text{-}688}$:DNA association using PEMSA. (A) The native polyacrylamide gel shows a decrease of fluorescent intensity when the concentration of inS3-54 is increased (log scale x-axis). **(B)** The DBD inhibitor inS3-54 shows an IC_{50} of ~26 μM.

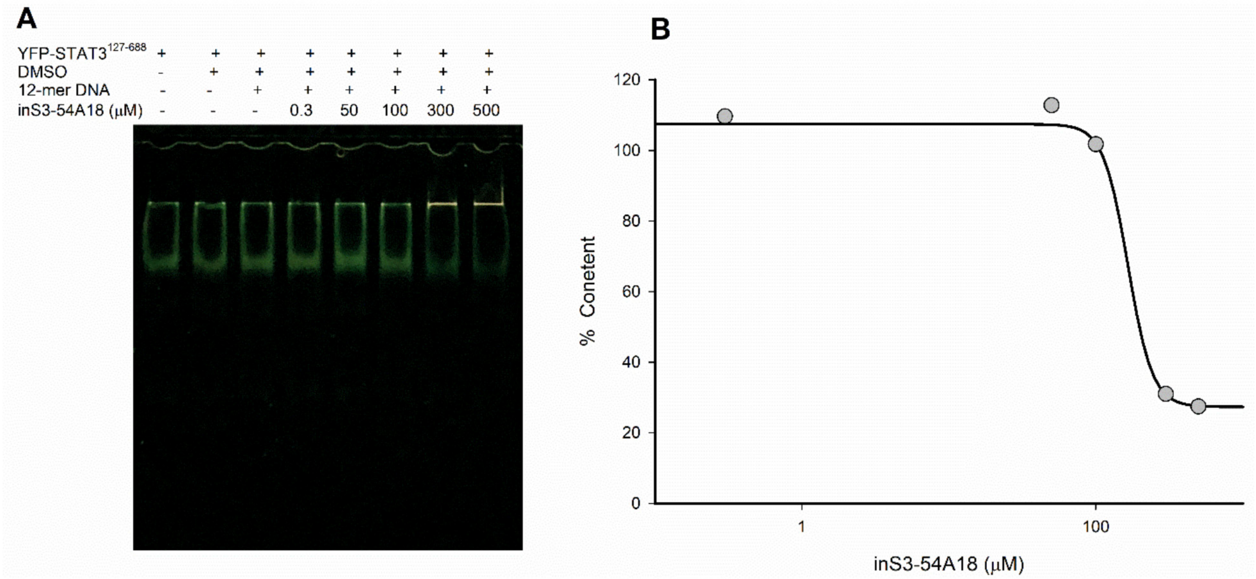

Supplementary Figure 3: Validation of the inhibitory effect of inS3-54A18 on YFP-STAT3$^{127\text{-}688}$:DNA association using PEMSA. (A) The native polyacrylamide gel shows a decrease of fluorescent intensity when the concentration of inS3-54A18 is increased (log scale x-axis). **(B)** inS3-54A18 shows an IC_{50} of ~165 μM.
